# Multimodality calibration for simultaneous fluoroscopic and nuclear imaging

**DOI:** 10.1186/s40658-016-0156-1

**Published:** 2016-08-30

**Authors:** Casper Beijst, Mattijs Elschot, Sandra van der Velden, Hugo W. A. M. de Jong

**Affiliations:** 1 Radiology and Nuclear Medicine, UMC Utrecht, P.O. Box 85500, 3508 GA Utrecht, the Netherlands; 2 Image Sciences Institute, UMC Utrecht, P.O. Box 85500, 3508 GA Utrecht, the Netherlands; 3 Department of Circulation and Medical Imaging, Faculty of Medicine, Norwegian University of Science and Technology, Trondheim, Norway

**Keywords:** Scintigraphy, c-arm, Fluoroscopy, Nuclear imaging, Interventional, Calibration, X-ray, Hybrid imaging, Dynamic imaging

## Abstract

**Background:**

Simultaneous real-time fluoroscopic and nuclear imaging could benefit image-guided (oncological) procedures. To this end, a hybrid modality is currently being developed by our group, by combining a c-arm with a gamma camera and a four-pinhole collimator. Accurate determination of the system parameters that describe the position of the x-ray tube, x-ray detector, gamma camera, and collimators is crucial to optimize image quality. The purpose of this study was to develop a calibration method that estimates the system parameters used for reconstruction.

A multimodality phantom consisting of five point sources was created. First, nuclear and fluoroscopic images of the phantom were acquired at several distances from the image intensifier. The system parameters were acquired using physical measurement, and multimodality images of the phantom were reconstructed. The resolution and co-registration error of the point sources were determined as a measure of image quality. Next, the system parameters were estimated using a calibration method, which adjusted the parameters in the reconstruction algorithm, until the resolution and co-registration were optimized. For evaluation, multimodality images of a second set of phantom acquisitions were reconstructed using calibrated parameter sets. Subsequently, the resolution and co-registration error of the point sources were determined as a measure of image quality. This procedure was performed five times for different noise simulations. In addition, simultaneously acquired fluoroscopic and nuclear images of two moving syringes were obtained with parameter sets from before and after calibration.

**Results:**

The mean FWHM was significantly lower after calibration than before calibration for 21 out of 25 point sources. The mean co-registration error was significantly lower after calibration than before calibration for all point sources. The simultaneously acquired fluoroscopic and nuclear images showed improved co-registration after calibration as compared with before calibration.

**Conclusions:**

A calibration method was presented that improves the resolution and co-registration of simultaneously acquired hybrid fluoroscopic and nuclear images by estimating the geometric parameter set as compared with a parameter set acquired by direct physical measurement.

**Electronic supplementary material:**

The online version of this article (doi:10.1186/s40658-016-0156-1) contains supplementary material, which is available to authorized users.

## Background

A major development in imaging for oncology during the last decades has been the development of hybrid imaging modalities such as PET/CT and SPECT/CT [1]. These systems are mostly used for diagnostic purposes, although PET/CTs have also been employed for image-guided (oncological) procedures [2]. However, the closed gantry geometry and the lack of real-time simultaneous imaging are suboptimal for interventional applications. Therefore, a modality for real-time simultaneous fluoroscopic and nuclear imaging is currently being developed by our group, by combining a c-arm and a gamma camera with a four-pinhole collimator [3].

Real-time simultaneous fluoroscopic and nuclear imaging has the potential to provide the physician with valuable information during interventional procedures. Examples of procedures that could benefit from real-time hybrid imaging are biopsies [4] and liver radioembolization [5].

Hybrid nuclear imaging modalities require a correct description of the acquisition geometry for accurate reconstruction and co-registration of images. In SPECT/CT, for example, these geometric parameters include mechanical offsets of the gamma cameras, electronic shifts, the rotation radius, and the relative position of modalities. Incorrect description of the SPECT/CT acquisition geometry can result in loss of resolution, image deformation, and co-registration errors, especially for pinhole and cone-beam collimators because of their magnifying properties [6–8]. The geometric parameters can be determined by direct physical measurement or by using calibration methods [9–11]. Calibration algorithms have also been employed for the correction and alignment of cone-beam CT images [12].

A correct description of the acquisition geometry is also required for the fluoroscopic and nuclear imaging c-arm, to prevent resolution loss, image deformation, and co-registration errors. The parameters that describe the geometry of the hybrid c-arm include the relative position of the gamma camera, collimators, x-ray tube, and x-ray detector (image intensifier). The purpose of this study was to develop a calibration method which improves the resolution and co-registration of simultaneously acquired hybrid fluoroscopic and nuclear images, by estimating the geometric parameter set used for reconstruction and co-registration using phantom measurements.

## Methods

### Prototype

**Fig. 1:**
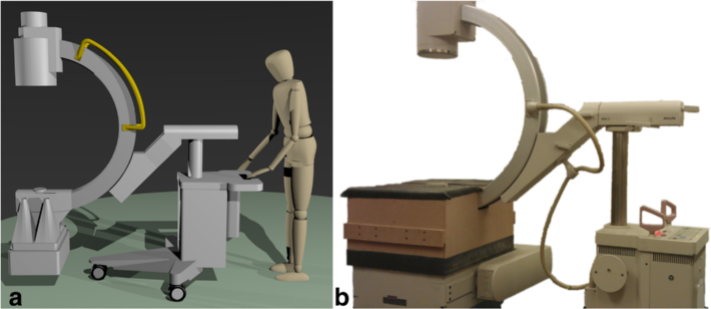
Rendering (**a**) and picture (**b**) of the interventional fluoroscopic and nuclear imaging system

Simultaneous fluoroscopic and nuclear imaging of the same field of view (FOV) is feasible when an x-ray tube, an x-ray detector, and a gamma camera with a four-pinhole collimator are placed in one line [3]. A prototype of this interventional fluoroscopic and nuclear imaging system was previously built using a Siemens Diacam gamma camera with a 9.5-mm NaI(Tl) scintillation crystal and a Philips BV29 c-arm with a 22.9-cm image intensifier (Fig. 1) [3]. A collimator with four 5-mm lead pinholes was especially designed to collimate gamma rays around the x-ray tube. At least 4 mm of lead shielding was applied around the four-pinhole collimator to ensure that uncollimated photons remained undetected.

### Nuclear image reconstruction

**Fig. 2:**
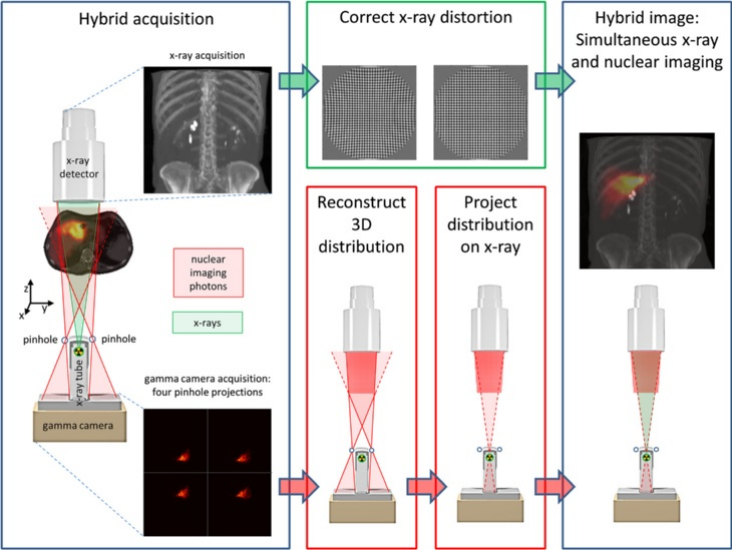
Flowchart showing steps involved in the post-processing of the acquired hybrid simultaneous images

A reconstruction algorithm was previously developed to create a nuclear image that overlapped with the fluoroscopic image, by conversion of the four-pinhole projections into a single image [3]. In short, the conversion of the four-pinhole projections involves two steps. First, the four projections are used to reconstruct a three-dimensional distribution with an iterative maximum likelihood expectation maximization (MLEM) algorithm [13]. The forward and backward projections of the reconstruction step incorporate resolution recovery by modeling the point response geometrically, taking into account the size of the pinhole opening and including penetration of the pinhole edges [14–16]. Second, the three-dimensional activity distribution is projected onto the x-ray detector by performing a cone-beam projection that is geometrically identical to the fluoroscopic projection. Hybrid fluoroscopic and nuclear images are subsequently obtained by showing the fluoroscopic image in grayscale and the nuclear image in color overlay. The process of creating hybrid fluoroscopic and nuclear images is shown schematically in Fig. 2.

### Fluoroscopic distortion correction

The fluoroscopic images were corrected for distortion effects of the image intensifier, before being overlapped with nuclear images. Distortion of fluoroscopic images as acquired by image intensifiers is a relatively common problem, and several strategies have been described in literature that involve the acquisition of a repetitive grid of features to estimate the position-dependent displacement [17–20].

**Fig. 3:**
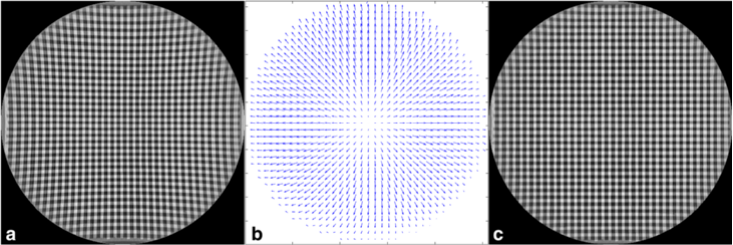
Fluoroscopic image of the lead grid before distortion correction (**a**), the acquired distortion matrix (**b**), and fluoroscopic image of the lead grid after distortion correction (**c**)

For correction, fluoroscopic acquisitions of a line-pattern phantom with 4-mm-wide strips of lead were performed, using a 45-kV tube voltage and a 0.2-mA tube current. The grid acquisition as shown in Fig. 3a was obtained by combining acquisitions of the lead grid in the *x* and *y* direction through subtraction. To obtain an image with enhanced grid points, a threshold was applied and binary erosion was performed, using in-house developed MATLAB (MathWorks Inc., Natick, MA, USA) code. Subsequently, the center of gravity of the enhanced grid points was calculated to determine their position. For each acquired grid point, the measured position was compared to the expected position. In this manner, the position-dependent grid point distortion in the *x* and *y* direction was determined (Fig. 3b). This was translated into a distortion matrix with the size of the fluoroscopic image for both the *x* and *y* direction by bi-linear interpolation. This distortion matrix was used to obtain corrected fluoroscopic images, as shown in Fig. 3b.

### Parameter calibration

To improve the resolution and co-registration of simultaneously acquired fluoroscopic and nuclear images, we developed a calibration method that estimates the geometric parameter set. An overview of the calibrated geometric parameters is given in Table 1. A correct estimate of the pinhole positions is required to prevent resolution loss of the nuclear image, whereas a correct estimation of the effective diameter of the x-ray detector was important for correct scaling of fluoroscopic images with respect to the nuclear images. The position of the x-ray detector was important for co-registration of fluoroscopic and nuclear images. To serve as an initial guess for the calibration, the geometric parameter set was determined by direct physical measurement. The pinhole positions were obtained using measuring tape. The x-ray tube and x-ray detector were placed in front of the center of the gamma camera as accurately as possible by visual inspection, to minimize the shift with respect to the center of the coordinate system. Consequently, the shift of the x-ray tube and detector with respect to the center of the gamma camera was initially assumed to be zero.

**Table 1:** Geometric parameters used by the co-registration and overlap algorithm

Symbol	Explanation
*x*_pinhole, *n*_	*x* coordinate of the pinhole nr. *n* = 1, 2, 3, 4
*y*_pinhole, *n*_	*y* coordinate of the pinhole nr. *n* = 1, 2, 3, 4
*d*_x-ray detector_	Effective diameter x-ray detector
*x*_x-ray detector_	Shift of the x-ray detector with respect to the center of the coordinate system
*y*_x-ray detector_	Shift of the x-ray detector with respect to the center of the coordinate system

**Fig. 4:**
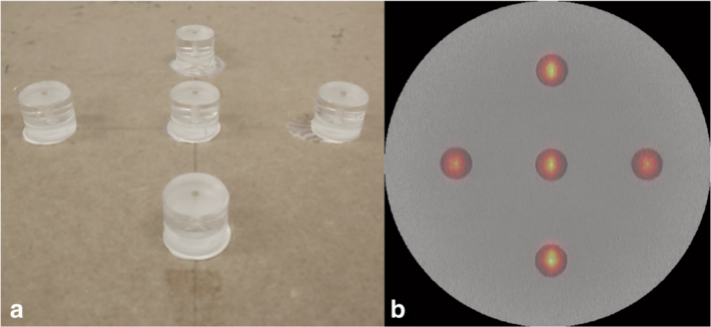
A picture of the five-point-source phantom (**a**) and a hybrid acquisition of the phantom showing the fluoroscopic image in grayscale and nuclear image in color overlay (**b**)

For calibration purposes, acquisitions were performed of a five-point-source phantom. The point sources were visible on both fluoroscopic and nuclear images and were created by filling a 3-mm spherical cavity in a polymethyl methacrylate cylinder with a diameter and thickness of 2 cm (Fig. 4). The five cavities were filled with 20, 25, 21, 21, and 17 MBq ^99m^Tc, respectively. The phantom was positioned at 4.7-, 10.7-, 16.7-, 22.7-, and 28.7-cm distances from the x-ray detector using a tape measure, resulting in a total of 25 point source positions used for calibration.

Fluoroscopic images were acquired with a 45-kV tube voltage and a 0.2-mA tube current. Nuclear images were acquired on 128 × 128 pixels per projection with 2.4 × 2.4 mm^2^ pixel size in 300 s per view. Reconstructions were performed with five MLEM iterations to obtain a volume of 128 × 128 × 128 voxels with a 2.4 × 2.4 × 2.4 mm^3^ voxel size for evaluation. The calibration required a computation time of approximately 3 h on a standard workstation.

**Fig. 5:**
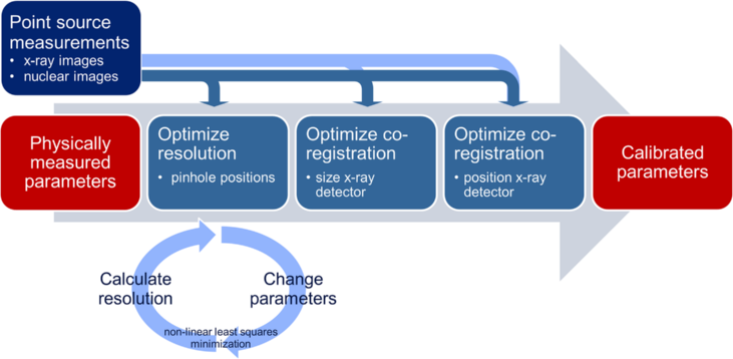
Schematic overview of the calibration procedure

A three-step calibration of resolution and co-registration was performed in MATLAB (MathWorks Inc., Natick, MA, USA) to obtain a calibrated parameter set, as explained below and as shown schematically in Fig. 5. As a measure of resolution, the full width at half maximum (FWHM) of the three-dimensional activity distribution in the *x* and *y* direction was determined. The FWHM was calculated by extracting profiles from the reconstructed activity distribution through the maximum voxel in the *x* and *y* direction, which were fitted with a Gaussian. The positions of the point sources on fluoroscopic and nuclear images were determined by applying a threshold and subsequent calculation of the centers of gravity. The co-registration error was determined by calculating the distance between the centers of gravity of the fluoroscopic and nuclear images as a measure of fiducial registration error (FRE).

#### Step 1: calibration pinhole positions

First, the resolution of the nuclear image was optimized by performing reconstructions for varying *x* and *y* components of the pinhole positions, and the resulting resolution was determined for each pinhole configuration. A non-linear trust-region-reflective sum-of-squares minimization algorithm was used to determine the optimal pinhole positions in terms of phantom resolution [21, 22]. Trust regions were used to prevent local convergence because in exploratory experiments not presented in this study, we observed that local convergence was likely to occur without the use of trust regions. The cost function used for the minimization of the resolution in the *x* and *y* direction was defined as

$$ {L}_{\mathrm{res}\ x,y}=\overline{{\mathrm{FWHM}}_{x,y}} $$

where $\overline{{\mathrm{FWHM}}_{x,y}}$ is the mean FWHM of the point sources in the *x* and *y* direction, respectively.

#### Step 2: calibration effective size x-ray detector

Once the optimal pinhole positions were found, the effective diameter of the x-ray detector was estimated, to improve the co-registration. The effective diameter was defined as the cross section of the image intensifier that was actually used for fluoroscopic imaging, which determined the effective diameter of the fluoroscopic images after correction. Therefore, the distance between the upper and lower point sources and the distance between the left and right point sources were calculated for both fluoroscopic and nuclear images. The effective diameter was scaled such that the mean of these distances over all positions of the phantom were equal.

#### Step 3: calibration position x-ray detector

Next, the mean shift between fluoroscopic and nuclear images was corrected for by incorporating the position of the x-ray detector in the *x* and *y* direction. The position of the x-ray detector was estimated by calculating the mean shift between point sources on fluoroscopic images and nuclear images in the *x* and *y* direction.

#### Evaluation

After calibration, the resolution of the nuclear images and the multimodality co-registration error were evaluated with a different set of point source measurements, to avoid using the same measurements for calibration and evaluation. For evaluation, the phantom was positioned at 1.7-, 7.7-, 13.7-, 19.7-, and 25.7-cm distances from the x-ray detector, resulting in a total of 25 point source positions used for evaluation. Subsequently, the mean resolution and the mean co-registration error as a measure of the target registration error (TRE) were determined at each distance. This was done for a geometric parameter set that was acquired by direct physical measurement and geometric parameter sets estimated using the calibration method.

The analyses described above were performed five times to assess the influence of noise on the calibration results. Therefore, additional measurements were simulated by adding Poisson noise to the measured projections. The mean and standard deviations of the resolution and co-registration error were determined, and the Standard Equivalency Test (SET) was used to determine whether the observed differences were significant. The SET is described as follows:

$$ \left|a-b\right|>2\sqrt{\sigma_a^2+{\sigma}_b^2} $$

where *a* and *b* are values measured with uncertainties *σ*_*a*_ and *σ*_*b*_, respectively.

### Simultaneous fluoroscopic and nuclear acquisition

To illustrate the co-registration of simultaneously acquired fluoroscopic and nuclear images in a dynamic situation, acquisitions were performed of two syringes containing 49 MBq of ^99m^Tc. The syringes were connected via a three-way valve, and the activity was flowing from one syringe into the other during the acquisition. Post-processing was performed twice, using a geometric parameter set that was acquired by direct physical measurement and using a geometric parameter set estimated by the calibration method.

Nuclear images were acquired with a frame rate of 2 frames per second. Reconstructions were performed with five MLEM iterations. Fluoroscopic images were acquired with a 43-kV tube voltage and a 0.14-mA tube current at 25 frames per second. Hybrid fluoroscopic and nuclear acquisitions were visualized at 5 frames per second. The nuclear images were super-sampled by means of interpolation for visual purposes to match the spatial and temporal matrix sizes of the visualized x-ray images [3, 23].

## Results

### Parameter calibration

**Fig. 6:**
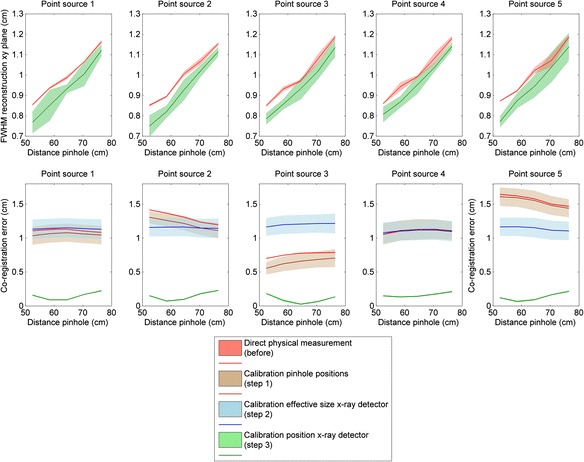
Graphical representations of the calibration results showing the resolution of the reconstructed volume in the *xy* plane and the co-registration error as a function of distance from the pinholes for the parameter set obtained by direct physical measurement, after calibrating the pinhole positions (step 1), after calibrating the effective size of the x-ray detector (step 2), and after calibrating the position of the x-ray detector (step 3) for the five point sources. The *shaded area* denotes the range between the mean plus two standard deviations and the mean minus two standard deviations

Table 2 shows the parameter values before, during, and after calibration. Figure 6 shows the FWHM of the reconstructed activity distribution in the *xy* plane (perpendicular to x-ray beam) as a function of distance from the pinholes. The mean FWHM was significantly lower for the parameter set estimated by the calibration method than for the parameter set acquired by direct measurement for 21 out of 25 point sources, as shown in Table 3. As expected, the resolution was improved by the resolution calibration (step 1) and remained unchanged during the subsequent calibration steps.

**Table 2:** Parameter values before, during, and after calibration

Parameter	Pinhole nr.	Direct physical measurement (before)	Calibration pinhole positions (step 1)	Calibration effective size x-ray detector (step 2)	Calibration position x-ray detector (step 3)
*x*_pinhole_ (cm)	1	−5.40	−5.527 ± 0.001	*	*
	2	−5.50	−5.488 ± 0.020	*	*
	3	5.40	5.291 ± 0.020	*	*
	4	5.50	5.551 ± 0.005	*	*
*y*_pinhole_ (cm)	1	−8.90	−8.810 ± 0.007	*	*
	2	8.90	8.911 ± 0.020	*	*
	3	−9.15	−9.094 ± 0.036	*	*
	4	9.15	9.057 ± 0.049	*	*
*d*_x-ray detector_ (cm)		22.86	22.86	21.39 ± 0.02	*
*x*_x-ray detector_ (cm)		0.00	0.00	0.00	−1.14 ± 0.07
*y*_x-ray detector_ (cm)		0.00	0.00	0.00	0.16 ± 0.03

The center of the coordinate system was the center of the scintillation crystal surface. The *z* axis was perpendicular to the scintillation crystal surface and pointed towards the x-ray detector

Asterisks (*) indicate that the parameter value was unchanged with respect to the previous calibration step

**Table 3:** Mean and standard deviation of the FWHM for the parameter set obtained before and after calibration

	Pinhole distance (cm)	1	2	3	4	5
Mean FWHM ± *σ*_FWHM_before calibration (cm)	52.4	0.854 ± 0.002	0.851 ± 0.003	0.850 ± 0.003	0.859 ± 0.001	0.872 ± 0.001
	58.4	0.937 ± 0.003	0.895 ± 0.002	0.932 ± 0.006	0.943 ± 0.01	0.923 ± 0.003
	64.4	0.988 ± 0.005	1.005 ± 0.006	0.971 ± 0.004	0.993 ± 0.004	1.022 ± 0.008
	70.4	1.065 ± 0.001	1.069 ± 0.008	1.079 ± 0.012	1.083 ± 0.014	1.072 ± 0.016
	76.4	1.163 ± 0.005	1.154 ± 0.004	1.187 ± 0.005	1.180 ± 0.006	1.187 ± 0.006
Mean FWHM ± *σ*_FWHM_after calibration (cm)	52.4	0.768 ± 0.027	0.749 ± 0.027	0.785 ± 0.014	0.807 ± 0.019	0.770 ± 0.014
	58.4	0.852 ± 0.038	0.821 ± 0.015	0.858 ± 0.013	0.868 ± 0.014	0.875 ± 0.019
	64.4	0.933 ± 0.011	0.926 ± 0.026	0.933 ± 0.026	0.953 ± 0.022	0.944 ± 0.027
	70.4	1.005 ± 0.030	1.023 ± 0.015	1.018 ± 0.020	1.039 ± 0.011	1.039 ± 0.037
	76.4	1.121 ± 0.014	1.115 ± 0.012	1.135 ± 0.024	1.141 ± 0.011	1.138 ± 0.034
$$ \begin{array}{l}\left|{\mathrm{FWHM}}_{\mathrm{before}}\right.\\ {}\left.-{\mathrm{FWHM}}_{\mathrm{after}}\right|\\ {}>\\ {}\sqrt[2]{\sigma_{\mathrm{before}}^2+{\sigma}_{\mathrm{after}}^2}\end{array} $$	52.4	Yes	Yes	Yes	Yes	Yes
	58.4	Yes	Yes	Yes	Yes	Yes
	64.4	Yes	Yes	No	No	Yes
	70.4	Yes	Yes	Yes	Yes	No
	76.4	Yes	Yes	Yes	Yes	No

Figure 6 also shows the co-registration error or TRE as a function of distance from the pinholes. The mean co-registration error was significantly lower for the parameter set estimated by the calibration method than for the parameter set acquired by direct physical measurement for all point sources, as shown in Table 4. The spread of the co-registration error was reduced when the effective size of the x-ray detector was calculated, whereas the mean co-registration error was reduced significantly after calibrating the position of the x-ray detector, as shown in Fig. 6.

**Table 4:** Mean and standard deviation of the co-registration error (shift) for the parameter set obtained before and after calibration

	Pinhole distance (cm)	1	2	3	4	5
Mean shift ± *σ*_shift_before calibration (cm)	52.4	1.108 ± 0.004	1.416 ± 0.003	0.701 ± 0.003	1.055 ± 0.002	1.644 ± 0.005
	58.4	1.127 ± 0.005	1.365 ± 0.005	0.748 ± 0.002	1.103 ± 0.005	1.620 ± 0.004
	64.4	1.131 ± 0.002	1.310 ± 0.005	0.778 ± 0.004	1.117 ± 0.002	1.578 ± 0.004
	70.4	1.103 ± 0.002	1.233 ± 0.003	0.784 ± 0.005	1.120 ± 0.004	1.499 ± 0.004
	76.4	1.083 ± 0.003	1.197 ± 0.005	0.789 ± 0.005	1.096 ± 0.002	1.467 ± 0.005
Mean shift ± *σ*_shift_after calibration (cm)	52.4	0.160 ± 0.006	0.153 ± 0.006	0.184 ± 0.005	0.150 ± 0.006	0.123 ± 0.005
	58.4	0.092 ± 0.005	0.073 ± 0.006	0.082 ± 0.011	0.134 ± 0.005	0.067 ± 0.008
	64.4	0.092 ± 0.004	0.098 ± 0.005	0.027 ± 0.007	0.143 ± 0.004	0.093 ± 0.006
	70.4	0.171 ± 0.002	0.184 ± 0.006	0.066 ± 0.006	0.175 ± 0.007	0.167 ± 0.005
	76.4	0.225 ± 0.006	0.23 ± 0.005	0.136 ± 0.003	0.212 ± 0.008	0.216 ± 0.007
$$ \begin{array}{l}\left|{\mathrm{shift}}_{\mathrm{before}}\right.\\ {}\left.-\kern0.5em {\mathrm{shift}}_{\mathrm{after}}\right|\\ {}\kern3em >\\ {}\sqrt[2]{\sigma_{\mathrm{before}}^2+{\sigma}_{\mathrm{after}}^2}\end{array} $$	52.4	Yes	Yes	Yes	Yes	Yes
	58.4	Yes	Yes	Yes	Yes	Yes
	64.4	Yes	Yes	Yes	Yes	Yes
	70.4	Yes	Yes	Yes	Yes	Yes
	76.4	Yes	Yes	Yes	Yes	Yes

### Simultaneous fluoroscopic and nuclear acquisition

**Fig. 7:**
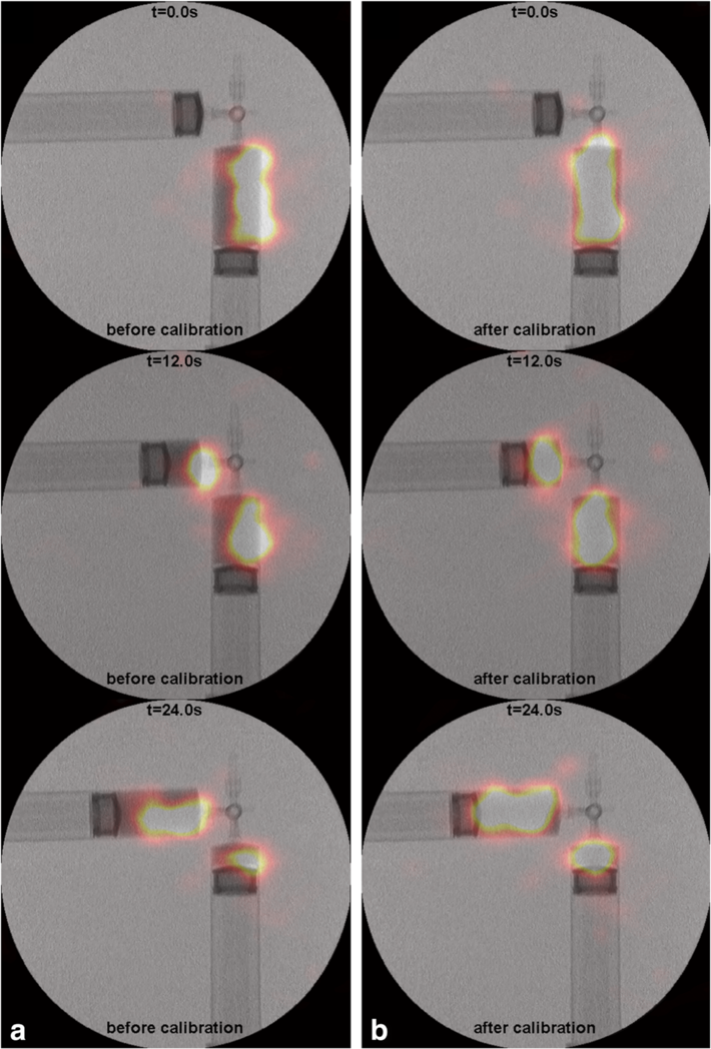
Montage of frames from a simultaneous hybrid acquisition (Additional file 1) of two syringes connected via a three-way valve filled with 49 MBq of ^99m^Tc showing fluoroscopic images in grayscale and nuclear images in color overlay. Images were obtained with a parameter set acquired by physical measurement (**a**) and with a parameter set acquired by the calibration method (**b**)

The simultaneously acquired dynamic fluoroscopic and nuclear images of the two syringes filled with 49 MBq of ^99m^Tc are shown in Fig. 7 and Additional file 1. With good spatial and temporal overlap of both modalities, a montage of fluoroscopic images is shown in grayscale and nuclear images in color overlay. Visual inspection of the images clearly showed an improvement of the co-registration of fluoroscopic and nuclear images by the calibration method. Differences in resolution of the nuclear images were difficult to assess visually.

Additional file 1: The simultaneously acquired dynamic fluoroscopic and nuclear images of the two syringes filled with 49 MBq of 99mTc. (MP4 5895 kb).

## Discussion

For hybrid fluoroscopic and nuclear imaging, a correct description of the acquisition geometry is required for accurate reconstruction and co-registration of images. A calibration method was presented that improved the resolution and co-registration of simultaneously acquired hybrid fluoroscopic and nuclear images by estimating the geometric parameter set as compared with a parameter set acquired by direct physical measurement.

**Fig. 8:**
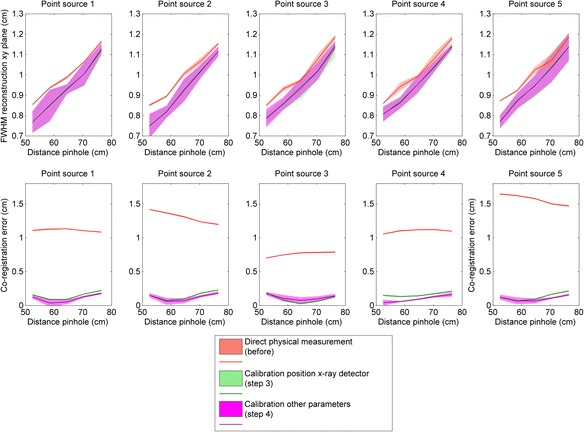
Graphical representations of the calibration results showing the resolution of the reconstructed volume in the *xy* plane and the co-registration error as a function of distance from the pinholes for the parameter set obtained by direct physical measurement (before), after calibrating the position of the x-ray detector (step 3) and after calibrating additional parameters (step 4) for the five point sources. The *shaded area* denotes the range between the mean plus two standard deviations and the mean minus two standard deviations

The remaining co-registration error after calibration was between 0 and 0.2 cm and may be reduced by optimizing additional geometric parameters in the calibration procedure using the Levenberg-Marquardt algorithm [24, 25]. However, as shown in Fig. 8 of the Appendix, the optimization of additional parameters had minor influence on the resolution and co-registration. Moreover, the amount of parameters used for calibration is a trade-off between accuracy and complexity. Model instability and computation time increase with complexity, and therefore, only the most important parameters were included in the calibration algorithm.

The construction of the experimental setup did not fully restrict movement of the c-arm with respect to the gamma camera. Therefore, a calibration of the system parameters was required each time the system was moved. Furthermore, the current experimental setup was only able to perform acquisitions in a single orientation. Future research aims to develop a prototype that can rotate around the patient, the same way conventional c-arms are used.

Corrections were required for the distortion of fluoroscopic images acquired using an image intensifier. Future research will involve the acquisition and development of a prototype with a digital flat-panel x-ray detector, capable of performing the first clinical studies. In this way, problems caused by distortion of image intensifiers will be overcome, since these problems are almost non-existent for digital flat-panel detectors.

Statistical analysis was performed to estimate the magnitude of type A errors by performing the analysis multiple times for different noise realizations. The magnitude of several sources of errors can also be estimated based on the knowledge of our system. That is, type B uncertainties that may affect the accuracy of the calibration include remaining errors due to non-linearities of the x-ray images. Several groups have shown that distortion correction can correct for non-linearities and reduce errors to well below 1 mm [18, 26, 27]. Other errors that may affect the accuracy of the calibration are the distortion and non-linearities of the gamma camera. These errors may explain the remaining co-registration error after calibration of up to 0.2 cm [28–30].

The resolution of the nuclear images depends on the number of iterations used during the reconstruction step. Five MLEM iterations were used during the reconstruction step of the overlap algorithm, which was found optimal for calibration and visualization purposes based on a visual trade-off between resolution and noise. As with all nuclear medicine examinations in clinical practice, the number of iterations (and/or subsets) is a trade-off between resolution and noise. For some tasks, high resolution is required and a large number of iterations are used, whereas images with low noise levels may be favored for other applications. More research needs to be done to determine the application-dependent optimal number of MLEM iterations for the fluoroscopic and nuclear imaging c-arm. The same holds for the frame rate of nuclear imaging. Guidance of interventional procedures may require high frame rates, whereas diagnostic imaging may require high image quality. The noise level of the acquired images will also depend on the amount of activity that is used, which in turn depends on the isotope and the procedure. Clinical experience will be required to optimize these parameters and is the subject of future research.

Limitations of the calibration method include that the co-registration error was only minimized for five positions on the image intensifier. The number of point sources could be increased to acquire calibration data for additional locations. However, an increased number of acquired datasets requires longer computation times and increases complexity.

Over the years, many different optimization algorithms have been developed and used for calibration of (hybrid) imaging modalities [7, 11, 31–34]. Using other optimization algorithms than the trust-region-reflective or Levenberg-Marquardt method used for the presented calibrations may affect computation times. However, the solution of the optimization is not expected to differ significantly for different optimization algorithms, as long as local convergence is avoided, which was ruled out by using trust regions.

The accuracy of the physical measurement was limited. For the pinhole positions, measuring tape was used, which resulted in an estimated measurement error of approximately 1 mm. Moreover, the shift of the x-ray detector and x-ray tube with respect to the gamma camera was minimized by visual alignment, which is error-prone. More accurate physical measurements could have been obtained using more advanced measurement equipment, such as laser distance meters. However, the authors believe that geometric parameters estimated by calibration can be obtained with high accuracy and that achieving the same accuracy by direct measurement would require expensive and advanced equipment. In addition, phantom measurements have the added advantage of allowing for easy evaluation of image quality.

The findings of this study emphasize the importance of correct system calibration for simultaneous fluoroscopic and nuclear imaging. Of course, the specific results are only applicable to the current prototype. The general methodology, however, will also be applicable to future prototypes of the hybrid c-arm and may also be of use for calibration of other hybrid modalities [35]. Therefore, the presented calibration algorithm is a crucial next step towards bringing real-time simultaneous fluoroscopic and nuclear imaging to the intervention room.

## Conclusions

A calibration method was presented that improved the resolution and co-registration of simultaneously acquired hybrid fluoroscopic and nuclear images by optimizing the geometric parameter set as compared with a parameter set acquired by direct physical measurement. The improvement of co-registration was verified qualitatively by hybrid imaging of a dynamic phantom.

## Appendix

The remaining co-registration error after calibration may be caused by not including some geometric parameters in the calibration procedure. Therefore, another calibration step was added to the calibration algorithm. This step incorporated additional calibration of the effective diameter of the image intensifier, the position of the image intensifier, and the position of the x-ray tube by using the Levenberg-Marquardt algorithm. The cost function used for the optimization was defined as

$$ {L}_{\mathrm{detector}\ \mathrm{position}}=\left|\overline{{\underset{x}{\to}}_{\mathrm{xray}} - {\underset{x}{\to}}_{\mathrm{nuclear}}}\right| $$

where ${\underset{x}{\to}}_{\mathrm{xray}}$ and ${\underset{x}{\to}}_{\mathrm{nuclear}}$ are the mean positions of the point sources on the x-ray images and nuclear images, respectively. In this case, the Levenberg-Marquardt algorithm was used because there was no need for trust regions, in contrast to the optimization of the pinhole positions.
